# Essex-Lopresti Fracture-Dislocation With Transolecranon Fracture-Dislocation: A Case Report

**DOI:** 10.7759/cureus.90041

**Published:** 2025-08-13

**Authors:** Ryo Kamiya, Kenji Onuma, Koji Sukegawa, Terumasa Matsuura, Masashi Takaso

**Affiliations:** 1 Department of Orthopaedic Surgery, Kitasato University School of Medicine, Sagamihara, JPN; 2 Research and Development Center for Medical Education, Kitasato University School of Medicine, Sagamihara, JPN

**Keywords:** achilles tendon allograft, elbow joint, essex-lopresti fracture-dislocation, high-energy injury, interosseous membrane injury, transolecranon fracture-dislocation

## Abstract

An Essex-Lopresti fracture-dislocation with transolecranon fracture-dislocation is a rare injury. This report describes surgical repair, including open reduction and internal fixation and repair of the interosseous membrane. A 66-year-old man fell from a height while rock climbing. He was diagnosed with a fracture-dislocation of his left elbow. He initially underwent an external fixation of the elbow joint. Open reduction and internal fixation of the olecranon and artificial radial head replacement were performed two weeks after the injury. Four months after the injury, the third surgery involved an ulnar shortening osteotomy, and an allogeneic Achilles tendon graft for interosseous membrane injury was performed. His Quick DASH (disabilities of the arm, shoulder, and hand) score was 2.27 points two years after the injury. This rare case of complicated upper arm injury was treated with multiple surgeries. We consider that the allogeneic Achilles tendon is one option as a material for interosseous membrane reconstruction.

## Introduction

A transolecranon fracture-dislocation is a trauma caused by direct external force on the dorsal side of the elbow in the mid-range flexion [1,2]. In this trauma, the trochlea of the humerus penetrates the olecranon, causing a comminuted intra-articular fracture of the olecranon and resulting in anterior displacement of the radial head and disruption of the ulno-humeral joint [1,2]. Unlike a Monteggia fracture-dislocation, the proximal radioulnar joint of the transolecranon fracture-dislocation is not dislocated [1-3].

While Essex-Lopresti fracture-dislocation typically occurs due to axial loading (e.g., falls onto an outstretched hand), leading to a triad of radial head fracture, distal radioulnar joint (DRUJ) dislocation, and rupture of the interosseous membrane of the forearm [4,5]. It is important to check the length of the ulna compared to the radius at the wrist. Ulnar-positive variance indicates that the ulna is longer than the radius, while ulnar-negative variance indicates that the ulna is shorter. An injury involving both a radial head fracture and ulnar-positive variance may be diagnosed as an Essex-Lopresti fracture-dislocation. Both transolecranon fracture-dislocation and Essex-Lopresti fracture-dislocation are rare injuries that typically result from high-energy trauma. The combination of transolecranon fracture-dislocation and Essex-Lopresti fracture-dislocation is unusual and challenging to manage, as these injuries are independently associated with poor functional outcomes if they are not acutely diagnosed and treated. As it often results from a high-energy trauma, it is necessary to pay attention to other complications such as soft tissue injury and other fractures.

Interosseous membrane rupture of Essex-Lopresti fracture-dislocation is often overlooked in the initial response and can lead to chronic pain and dysfunction if left untreated [4,5]. The interosseous membrane has five components. The central band is the most consistent structure and is both the widest and thickest part of the membrane. It runs obliquely and distally from the radius to the ulna at an angle of 20-24 degrees with respect to the long axis of the ulna [4]. Various tissues, including autologous, allogeneic, and artificial ones, have been used and reported as reconstruction materials for the interosseous membrane, especially the central band [4-9]. Here, we report a case of Essex-Lopresti fracture-dislocation with a transolecranon fracture-dislocation in which the interosseous membrane was reconstructed using allogeneic Achilles tendons following osteosynthesis and ulnar shortening osteotomy. To the best of our knowledge, this is the first case report of Achilles tendon allografting for an interosseous membrane injury involving combined fractures and dislocations.

## Case presentation

**Figure 1:**
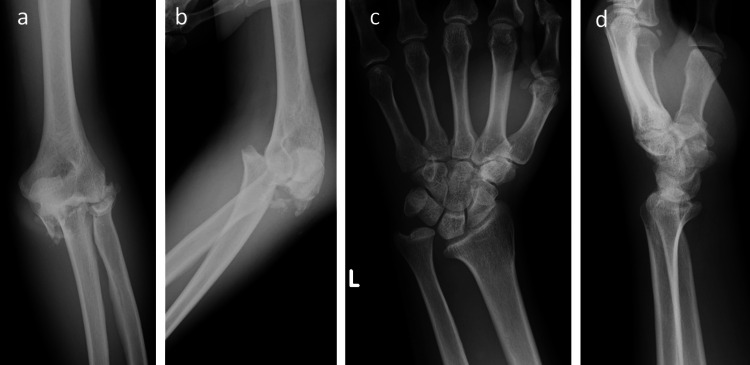
Preoperative X-ray images. (a) Anteroposterior and (b) lateral plain radiographs of the left elbow at initial presentation. These figures show a fracture-dislocation of the elbow. (c) Anteroposterior and (d) lateral radiographs of the left wrist. These figures show dislocation of the distal radioulnar joint.

The patient was a 66-year-old surgeon and an experienced mountaineer who slipped from a height of approximately 10 m while mountain climbing. He was transported to the emergency room and was diagnosed with anterior dislocation of the left shoulder joint, fracture of the left olecranon, and fracture of the radial head (Figure 1).

**Figure 2:**
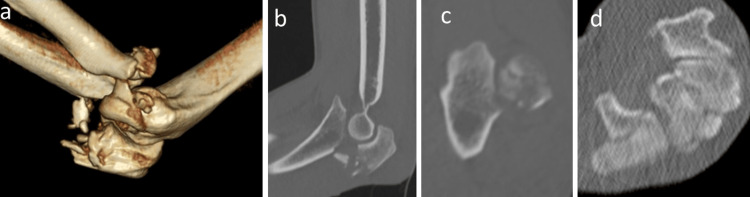
Preoperative CT. (a) Three-dimensional image of the left elbow. This figure shows fracture of the olecranon and radial head and dislocation of the humeroradial joint. (b) Sagittal plane of the left elbow. This figure shows intra-articular fractures of olecranon. (c) Axial plane of the left proximal radioulnar joint. This figure shows proximal radioulnar joint which is not dislocated. (d) Axial plane of the left carpal bone. This figure shows fracture of the hook of the hamate.

He returned home after the manipulation of the shoulder dislocation and splinting of the elbow joint. He visited our hospital the day following the trauma, presenting with swelling and blistering from his left elbow to his forearm with moderate pain, but without sensory impairment and circulatory disorder in his upper limbs. Plain X-ray images and CT (Figure 2) revealed a transolecranon fracture-dislocation, an Essex-Lopresti injury, a fracture of the hook of the hamate, and a rib fracture.

**Figure 3:**
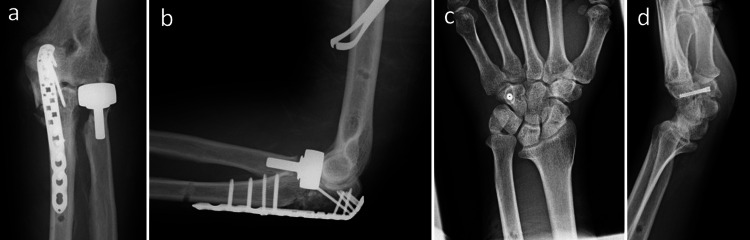
Plain X-ray image after the second surgery. (a) Anteroposterior and (b) lateral plain radiographs of the left elbow. Olecranon fracture was fixed with a locking plate. The fractured radial head was replaced with an artificial radial head. (c) Anteroposterior and (d) lateral plain radiographs of the left wrist. The non-displaced hook of the hamate fracture was fixed with a headless compression screw. The distal radioulnar joint remains dislocated after the second surgery.

On the day of the first visit to our hospital, following manipulation of the fracture-dislocation of the elbow, spanning external fixation across the elbow was performed under general anesthesia. Following the resolution of the swelling and blistering two weeks after the injury, a second surgery was performed. After the radial head with a comminuted fracture was removed using the posterior global approach to the elbow joint, it was replaced with an artificial radial head implant (Evolv; Wright Medical Technology, Inc. Arlington, TN), and the olecranon fracture was fixed with a locking plate, VA-LCP Olecranon plate (DePuySynthes, Raynham, MA). The undisplaced hamate hook fracture was treated percutaneously with a headless compression screw (Acutrak 2) via a palmar approach, minimizing soft-tissue disruption (Figure 3).

**Figure 4:**
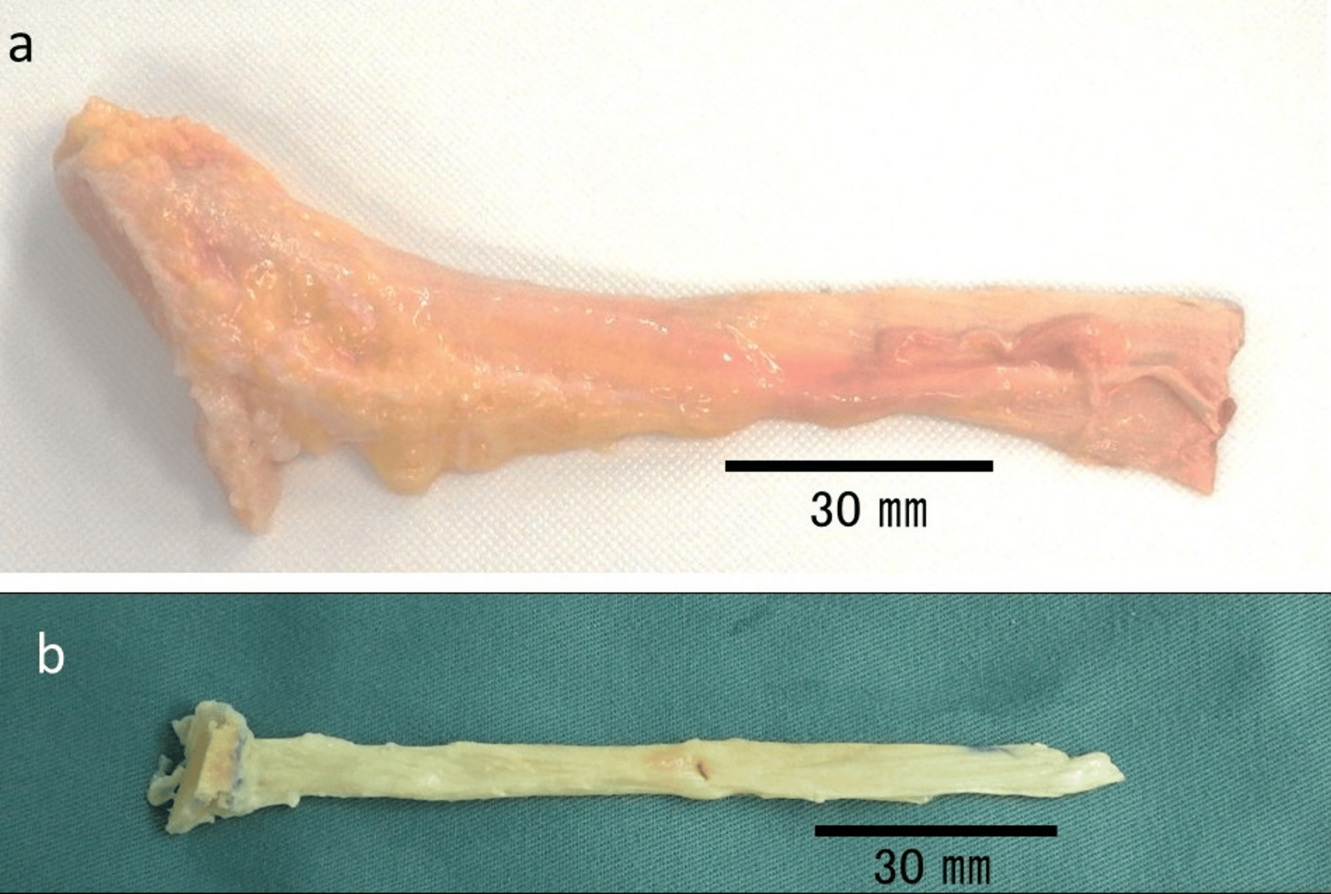
Allogenic Achilles tendon graft. (a) Allogenic Achilles tendon graft with partial calcaneal bone. (b) Trimmed Achilles tendon and calcaneal bone for the reconstruction of the interosseous membrane.

**Figure 5:**
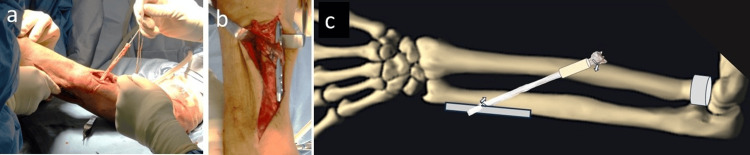
Intraoperative findings and schematic diagram. (a) Allogenic Achilles tendon graft passed through the bone tunnel of the radius. (b) Allogenic Achilles tendon graft fixed on the ulnar shaft. (c) A schematic diagram of the reconstructed central band. We used an application from teamLab Body to create this schematic diagram (URL for download: https://teamlabbodypro.onelink.me/WLCD/uapw0i47, and official website: https://www.teamlabbody.com/).

After achieving olecranon fracture union and seasonable elbow range of motion, the patient underwent a third surgical intervention four months post injury. Preoperative assessment demonstrated a residual flexion contracture of 35° (extension), with flexion to 125°, supination limited to 30°, and pronation to 80°. Radiographic evaluation revealed a pronounced ulnar-positive variance of 15 mm in the affected wrist, compared to 3 mm on the contralateral side. Chronic longitudinal radioulnar dissociation, confirmed by the observed 12 mm discrepancy in ulnar variance relative to the unaffected wrist, established the indication for central band reconstruction [2]. A radial proximal migration of >6 mm indicates injury to both the triangular fibrocartilage complex between the ulna and the radius intraoperatively in a manual test; possibly because of cicatricial contracture, we considered longitudinal instability due to rupture of interosseous membrane from the evidence of the significant ulnar-positive variance of 12 mm compared with the healthy side. We planned surgery to ensure that the ulnar variance of the left wrist was the same length as that of the healthy side and that the central band was reconstructed. A standardized lateral approach to the ulna was employed, with the limb suspended in finger traps to facilitate alignment. A 12 mm ulnar shortening osteotomy was performed using the Aptus Ulnar Shortening System 2.5 (Medartis AG, Switzerland). Due to the system’s 8 mm resection limit, the procedure was staged: an initial 8 mm resection was followed by plate repositioning and a subsequent 4 mm osteotomy. Following ulna shortening osteotomy, we performed reconstruction of the central band of the interosseous membrane. Via a posterior radial approach, a 5 mm osseous tunnel was created 160 mm distal to the radial styloid (approximately 60% of radial length). A #2 FiberLoop artificial ligament (Arthrex, Inc., Naples, FL) was threaded through the tunnel and secured to a trimmed allogeneic Achilles tendon graft using a locking tendon suture technique (Figures 4-5).

The graft was oriented at 25° relative to the ulnar longitudinal axis and affixed with manual maximum tensile strength to the ulnar plate without additional osseous tunneling (Figure 5). Both the radial and ulnar sides were reinforced with a single 1.4 mm soft anchor (JuggerKnot; Zimmer Biomet Holdings, Inc., Warsaw, IN). After these procedures, there was no instability of the distal radioulnar joint, so we did not reconstruct the triangular fibrocartilage complex. After surgery, sugar-tong forearm splinting was performed for three weeks, followed by range of motion training of the forearm and elbow.

**Figure 6:**
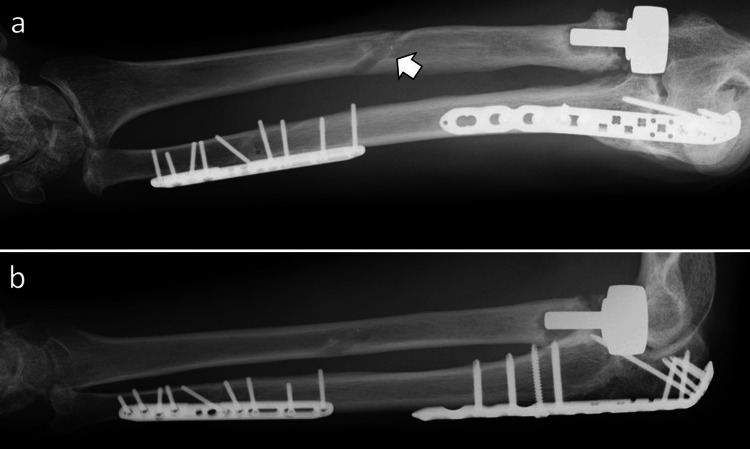
X-ray images at the final observation. (a) Anteroposterior and (b) lateral plain radiographs of the left forearm. Both the fracture and osteotomy site are united. An oblique bone tunnel can be seen in the shaft of the radius (white arrow).

At the most recent observation two years postoperatively, the grip strength of the right and left hands was 41 and 32 kg, respectively. The range of motion of the left elbow had improved to -10° in extension and 135° in flexion. The range of motion of the left forearm had improved to 70° in supination and 65° in pronation. The range of motion of the left wrist had improved to 45° in dorsal flexion and 40° in palmar flexion. On physical examination of his left wrist, the DRUJ ballottement test, ulnocarpal stress test, and ulnar foveal sign were negative. A Quick Disabilities of Arm, Shoulder and Hand score (DASH score) and a Japanese Orthopaedic Association-Japan Elbow Society Elbow Function Score (JOA-JES score) were 2.27 points and 89 points, respectively. Imaging revealed bone fusion at all fracture sites (Figure 6). The patient is recovering sufficiently to return to daily activities and reported no left elbow or wrist pain during surgery and mountaineering.

## Discussion

The Essex-Lopresti fracture-dislocation is a rare injury with an incidence of 1% of all radial head fractures [10]. Three main patterns of complex elbow instability have been described: valgus fracture-dislocation, well known as terrible triad, varus posteromedial in which anteromedial coronoid fracture with ligament complex disruption occurs, and transolecranon fracture dislocations [2]. Transolecranon fracture-dislocation is the least frequent pattern of injury after the other two [2]. Essex-Lopresti injury occurs when a high-energy load is axially applied to the forearm, usually due to a fall on an outstretched hand [4]. Transolecranon fracture-dislocation frequently occurs following a blow to the dorsal aspect of the forearm during mid-range flexion of the elbow [1,2]. This patient sustained an Essex-Lopresti fracture-dislocation due to landing on the palm of his left hand with the elbow extended during the fall. Immediately after that, he hit the olecranon on the ground with the elbow flexed, complicated by a transolecranon fracture-dislocation.

Although the best course for the treatment of this complicated injury was undefined, in the present case, we implemented a staged surgical approach that yielded favorable functional outcomes. The decision for delayed definitive fixation was necessitated by the presence of significant soft tissue compromise, including extensive swelling and blistering upon initial presentation. This cautious approach mitigated the risks of postoperative infection and compartment syndrome while allowing for soft tissue recovery prior to open reduction and internal fixation of the olecranon fracture. Moreover, as it is necessary to restrict the movement of the forearm and elbow with a postoperative sugar-tong splint for interosseous membrane reconstruction, we considered performing ulnar shortening osteotomy and reconstruction of the central band after recovering from both swelling and range of motion of the left arm.

There are various methods for interosseous membrane reconstruction. Grafting of autologous or allogenic bone-patellar tendon-bone (BPTB) [4] or autologous fascia lata [6] and rerouting of the pronator teres [4] or brachioradialis tendon [8] have been reported. Nonabsorbable braided sutures (TightRope; Stryker Corporation, Kalamazoo, MI), synthetic ligaments, and suture-and-button constructions have been reported as artificial ligaments [4].

The use of allogeneic Achilles tendon grafts for interosseous membrane reconstruction has been previously documented in cadaveric studies [8,9]. While biomechanical investigations have demonstrated that Achilles tendon grafts provide approximately 50% of the force transfer capacity compared to native ligamentous tissue [8], clinical outcome data remain limited. In the present case, we selected an allogeneic Achilles tendon graft based on several considerations: (1) its established utility in ligamentous reconstructions of other major joints (shoulder, knee, and ankle) at our institution and (2) the technical advantages afforded by its adaptable thickness and tensioning properties through selective trimming.

On the other hand, artificial ligaments have the potential advantages of being ready-to-use, lower cost, and lower risk of disease transmission compared to the allograft. The latter is extremely rare with modern allograft processing techniques. Gaspar et al. [11] reported on the reconstruction of the interosseous membrane using an artificial ligament, the Mini TightRope. In their report, three of the 10 patients reported complications requiring additional surgery. Three patients required additional surgery after undergoing interosseous membrane reconstruction with the TightRope technique. One patient complained of persistent ulnar impingement symptoms, which were possibly due to inadequate shortening during the initial surgery. This patient's symptoms improved with an additional ulnar shortening osteotomy. The second patient sustained a radial shaft fracture. The fracture occurred at the radial bone tunnel of the Mini TightRope. The patient underwent open reduction and internal fixation of the fracture, which healed without incident. The final patient complained of extreme difficulty with forearm supination. After removing the TightRope, they performed a closing ulnar wedge osteotomy. We speculate that the complications in the latter two cases occurred because the TightRope is less elastic than an allograft.

As the postoperative course was good, we conclude that this procedure is effective using the Achilles tendon allograft as an option for the reconstruction of the interosseous membrane. Given the single-case nature of this report, statistical analysis was not performed. As the clinical outcome of an Achilles tendon allograft for interosseous membrane reconstruction remains unknown, we need to observe clinical outcome carefully for a longer period of more patients.

## Conclusions

We experienced one rare case of Essex-Lopresti fracture-dislocation complicated by a transolecranon fracture-dislocation. The operation was performed in three stages. We first performed external fixation of the elbow, then open reduction and internal fixation of the elbow with radial head replacement, and finally reconstruction of the central band of the forearm interosseous membrane with Achilles tendon allograft and ulna shortening osteotomy. Good results were obtained, although there was still a limitation in the range of motion of the elbow and forearm. Although various tissue types have been used for interosseous membrane reconstruction, the allogeneic Achilles tendon is an effective reconstruction material.
